# Short- and Long-Term Study of the Impact of Focal Blue Light-Emitting Diode-Induced Phototoxicity in Adult Albino Rats

**DOI:** 10.3390/ijms22189742

**Published:** 2021-09-09

**Authors:** Juan A. Miralles de Imperial-Ollero, Alejandro Gallego-Ortega, María Norte-Muñoz, Johnny Di Pierdomenico, José Manuel Bernal-Garro, Francisco J. Valiente-Soriano, Manuel Vidal-Sanz

**Affiliations:** Departamento de Oftalmología, Universidad de Murcia e Instituto Murciano de Investigación Biosanitaria (IMIB) Virgen de la Arrixaca, Campus de CC de la Salud, 30120 El Palmar, Murcia, Spain; juanantoniomiralles@gmail.com (J.A.M.d.I.-O.); alejandrogallego@um.es (A.G.-O.); maria.norte@um.es (M.N.-M.); johnnydp@um.es (J.D.P.); jmbg@um.es (J.M.B.-G.)

**Keywords:** LED-induced phototoxicity, microglia activation, cone photoreceptor, adult albino rat

## Abstract

Background: In adult rats we study the short- and long-term effects of focal blue light-emitting diode (LED)-induced phototoxicity (LIP) on retinal thickness and Iba-1^+^ activation. Methods: The left eyes of previously dark-adapted Sprague Dawley (SD) rats were photoexposed to a blue LED (20 s, 200 lux). In vivo longitudinal monitoring of retinal thickness, fundus images, and optical retinal sections was performed from 1 to 30 days (d) after LIP with SD-OCT. Ex vivo, we analysed the population of S-cone and Iba-1^+^ cells within a predetermined fixed-size circular area (PCA) centred on the lesion. Results: LIP resulted in a circular focal lesion readily identifiable in vivo by fundus examination, which showed within the PCAs a progressive thinning of the outer retinal layer, and a diminution of the S-cone population to 19% by 30 d. In parallel to S-cone loss, activated Iba-1^+^ cells delineated the lesioned area and acquired an ameboid morphology with peak expression at 3 d after LIP. Iba-1^+^ cells adopted a more relaxed-branched morphology at 7 d and by 14–30 d their morphology was fully branched. Conclusion: LIP caused a progressive reduction of the outer retina with loss of S cones and a parallel dynamic activation of microglial cells in the lesioned area.

## 1. Introduction

One of the most severe ocular pathologies in the elderly is age-related macular degeneration (AMD), which is characterised by a progressive alteration of the resident cones in the macula, which produces gradual alterations in central vision and, in many cases ends up leading to blindness [1,2]. Although this disease has been extensively studied, its aetiology is not totally defined. The main risk factors for the development of AMD include age as the main factor, smoking, hypertension, or obesity [3,4]. However, it has been documented that there are other environmental factors that may favour the onset of this disease, such as exposure to light [5,6,7]. Because of this, many animal studies have focused on the study of the degeneration process of photoreceptors, both in inherited models [8,9,10,11,12,13] and in models of induced phototoxicity [5,14,15,16,17,18]. In the development of phototoxicity induction models, many recent studies have used light-emitting diode (LED) sources that show a deleterious effect of the light with an involvement of the retina, more aggressive in the outer region affecting photoreceptors and retinal pigment epithelium (RPE) cells [15,18,19,20,21,22,23,24]. In our laboratory we have developed an animal model of blue LED-induced focal phototoxicity (LIP) that causes a well-defined focal lesion within the superior-temporal region of the retina, where L-cone and retinal ganglion cell densities are highest [25,26] affecting mainly the outer retina and leading to photoreceptor degeneration. An alteration of the RPE cells has also been documented in other studies of LIP [23,27]. Degeneration and death of photoreceptors induced by LED phototoxicity have been shown to be triggered by apoptosis [18,20,28,29,30,31], although necrosis due to energy condensation has also been described [29,32]. In this model, the light insult also causes the alteration of the microglia that is activated and concentrated in the area of the lesion, and which has been characterised in the short term after LIP [18]. This activation has been already documented [27,30,31] and is characterised by a migration of microglial cells that change their morphology from their resting branching pattern to an ameboid shape a few days after induction of phototoxicity and progressively return to a branching pattern [18]. However, to the best we know, there are no long-term studies of the status of microglia after blue light induced retinal phototoxicity.

The aim of this work is to study in the same rat retinas: (i) in vivo, the short- and long-term effects of focal phototoxicity induction with blue light on inner and outer retinal thickness, and (ii) ex vivo, the loss of S cones and the chronology of the activation of microglial cells located in the lesioned area.

## 2. Results

### 2.1. Short- and Long-Term In Vivo Monitoring of Focal LED-Induced Phototoxic Retinal Damage

**Figure 1:**
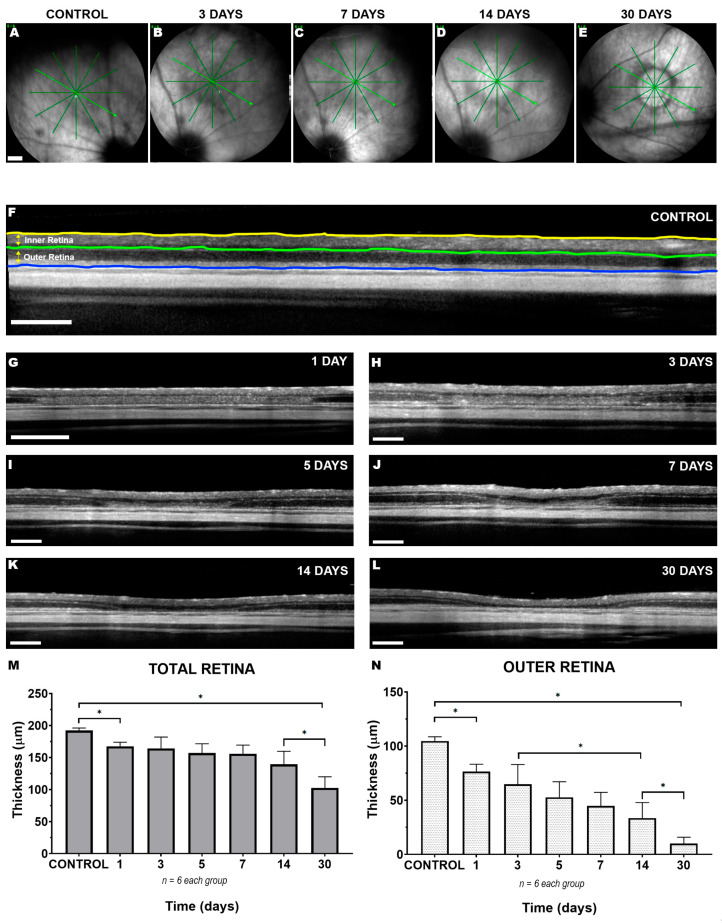
In vivo SD-OCT study of short- and long-term retinal thickness evolution after LIP induction. Representative eye fundus images acquired with infrared filter from a non-photoexposed control eye (**A**) and photoexposed experimental eyes at 3 (**B**), 7 (**C**), 14 (**D**), or 30 (**E**) days after LIP. For the study of total retinal thickness (from the fibre layer to the retinal pigment epithelium) and outer retinal thickness (from the outer plexiform layer to the retinal pigment epithelium) (**F**) at the centre of the lesion, it was measured using the mean of three calliper measurements provided directly by the device software. Panels (**G**–**L**) show representative images of lesion evolution at days 1, 3, 7, 14, and 30 after LIP. Total and outer retinal thickness measurements were analysed, and their graphical representations are shown in panels (**M**,**N**), respectively. *n* = 6 per group; * *p* < 0.05; (Kruskal–Wallis test).

Spectral Domain Optical Coherence Tomography (SD-OCT) fundus imaging showed the blue LED-induced focal lesion consistently located in the superior-temporal region of the experimental left retinas. This lesion became apparent 24 h after LIP, showing a definite circular decolorated area (Figure 1A–E). The lesion progressed over the following days, the discoloration became more evident in the centre of the lesion and the border became more clearly defined, reaching maximum expression 30 days after LIP injury (Figure 1E). The study of retinal thickness in the centre of the lesion by acquisition of SD-OCT optical sections from 1 to 30 days after LIP showed a progressive reduction of the retina that mainly affected the outer retina (Figure 1F–L). Six right fellow non-photoexposed retinas from the group analysed at 30 days were used as controls, and these had a total retinal thickness average of 192.3 ± 3.8 µm. In the LIP retinas, total central thickness was reduced by 1 day (167.6 ± 6.3 µm, *n* = 6; *p* = 0.075; Tukey Test) with no further significant reduction until 14 days (139.4 ± 20.4 µm, *n* = 6; *p* = 0.07; Tukey Test) and with further significant thinning by 30 days (102.8 ± 17.2 µm, *n* = 6; *p* < 0.001; Tukey Test) (Figure 1M). Detailed study of the central thickness of the inner retina showed a slight increase at 5 days after LIP (87.4 ± 2.2 µm to 104.0 ± 4.6 µm, *n* = 6; *p* < 0.05; Kruskal–Wallis), which returned to normal values at 30 days (92.9 ± 12.9 µm, *n* = 6). Regarding the evolution of the thickness of the outer retina, 1 day after LIP it was already significantly reduced (104.7 ± 3.9 µm to 76.4 ± 6.9 µm, *n* = 6; *p* = 0.004; Kruskal–Wallis). This reduction was continuous throughout all the period of the study so that by 30 days its mean thickness reached 10.2 ± 5.7 µm (*p* < 0.0001; Kruskal–Wallis) (Figure 1N).

### 2.2. Long-Term Study of Microglial Activation within the Damaged Focal Region

**Figure 2:**
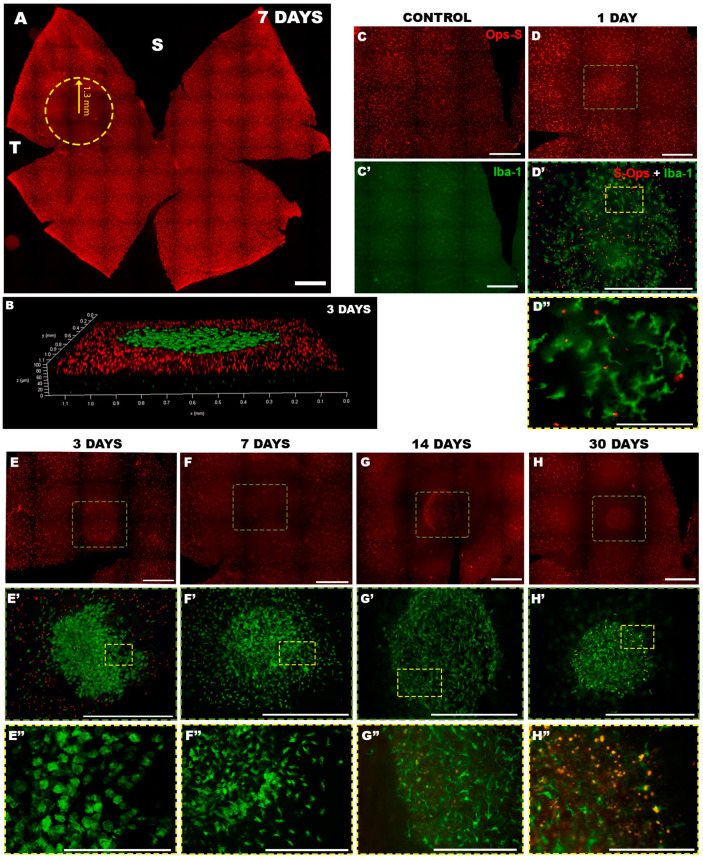
Progressive S-cone loss parallels activation of Iba-1^+^ cells in the focal area of lesions after LIP. LIP results in a progressive loss of S cones in a focal injured area located in the superior-temporal retina (**A**). This loss was accompanied by activation of Iba-1^+^ cells within the lesion area in the OS layer (**B**). Panels (**C**–**H’’**) show the status of S cones (labelled with Opsin S) and Iba-1^+^ cells in the OS layer in a non-photoexposed control fellow retina (**C**,**C’**) and in photoexposed experimental retinas analysed at days 1 (**D**–**D’’**), 3 (**E**–**E’’**), 7 (**F**–**F’’**), 14 (**G**–**G’’**), or 30 (**H**–**H’’**) after LIP. S: superior; T: temporal. Scale bar in A = 1 mm. Scale bar in (**C**–**H’**) = 500 µm. Scale bar in (**E’’**–**H’’**) = 100 µm.

As it is typical of this model, retinal wholemounts consistently showed a phototoxic lesion in the superior-temporal quadrant in all the left experimental eyes (Figure 2A). To monitor the evolution of phototoxic damage and to correlate the activation of microglia in the OS layer with the loss of cones, the population of S cones within the predetermined fixed-size circular area (PCA) was analysed and quantified (Table 1). S-opsin^+^ outer segments (S-opsin^+^OS) counts within the non-photoexposed right PCAs of the groups analysed at days 1 and 30 after LIP had similar values (2321 ± 435 and 2477 ± 357, respectively; *n* = 6 per group; with no significant differences, *p* = 0.512; *t*-test). Therefore, we pooled the S-opsin^+^OS counts of the right PCAs (1540 ± 236) to compare with the S-opsin^+^OS counts of the photoexposed left PCAs (Table 1). In the photoexposed left PCAs, the S-opsin^+^OS counts showed a progressive reduction that was first significant by 1 day after LIP when compared to S-opsin^+^OS counts in PCA of fellow right control retinas (1369 ± 364 vs. 2398 ± 435; *n* = 6 and 12 per group, respectively; *p* < 0.0001; one-way ANOVA). S-opsin^+^OS reduction continued until 14 days after LIP (361 ± 129; *n* = 6) (*p* = 0.002; one-way ANOVA) without further significant loss by 30 days after LIP (445 ± 67; *n* = 6) (*p* = 0.96; one-way ANOVA).

Our present results were compared with those obtained previously in albino rats from our group [15] to assess the similarity of the lesion. Indeed, there were no differences between our results and those of Ortín-Martínez et al. (2014) [15] at 7 days after LIP (795 ± 283 vs. 581 ± 211; S-opsin^+^OS, respectively; *p* = 0.13; *t*-test) confirming the homology of the model. Nevertheless, our S-opsin^+^OS counts at 30 days were smaller than those provided previously (445 ± 67 vs. 627 ± 71 S-opsin^+^OS, respectively; *p* = 0.001; *t*-test), a finding that may be explained by the fact that we have used in the present studies a rather longer exposure time of blue light (20 s in the present studies vs. 10 s in the former).

The short- and long-term study of microglial cell activation in the OS layer (Figure 2B) showed a clear involvement from day 1 after LIP which was maintained until 30 days (Figure 2C–H’’). Typical microglia cells residing in the OS layer of a control right retina showed the characteristic branched pattern of these cells which were distributed homogeneously throughout this layer (Figure 2C,C’). However, as soon as 1 day after LIP, activated microglial cells began to be observed delineating the light-damaged area (Figure 2D–D’’), in parallel with the reduction of S-opsin^+^OS. In terms of morphology, microglial cells in the lesioned area had different characteristics to those in the non-photoexposed retinas; the former had spindle-shaped somas and as a differentiating characteristic they showed a retraction of their primary processes with the emergence of short and fine processes directly from the cell soma. 

By 3 days, the lesioned area had progressed with an evident loss of S-opsin^+^OS together with a colocalisation of an increased number of microglial cells in the centre of the lesion. These microglial cells appeared with well-rounded cell bodies containing vacuoles, from which multiple short, thin, processes emerged, giving the cell a hairy appearance (Figure 2E–E’’). At the periphery of the lesion, there were microglial cells with a spindle-shaped soma, with more elongated extensions than those seen in the centre of the lesion. 

Cone loss progressed by 7 days after LIP and by 14 days an almost total loss of S-opsin^+^OS was observed in the centre of the lesion. In parallel, microglial cells remained accumulated within the area of lesion but showed changes in their morphology. At 7 days the microglia were characterised by a progressive change towards a branching morphology similar to the initial times of the study (Figure 2F–F’’). Microglial cells presented a more elongated and thicker soma that decreased at 14 days after LIP (Figure 2G–G’’). However, at the periphery of the lesion, cells with a more rounded soma were still observed with vacuoles inside the soma, from which prolongations arose, equivalent to those described at 5 days. At this time some autofluorescent deposits, which were visible under different fluorescent filters, started to be observed sometimes within the somas of the microglial cells. This autofluorescent material probably corresponds to cellular detritus or pigments that may or may not be integrated into macrophages and/or microglial cells. By 30 days after LIP, an almost total decrease of microglial cells was observed in the OS layer within the lesioned area together with loss of S-opsin^+^OS, and the autofluorescent material became more evident throughout the lesion area (Figure 2H–H’’). By this time, the morphology of the microglia stabilised in its branched form with a spindle-shaped soma from which multiple primary processes project, similar to that examined at 14 days.

## 3. Discussion

This study shows in vivo and ex vivo short- and long-term retinal alterations after induction of focal phototoxicity with a blue LED. LIP produces a progressive decrease in retinal thickness in the centre of the lesion that affects to a greater extent the outer retina and progresses until 30 days post lesion; during this period the total retinal thickness is reduced to 53.5% while the outer region is reduced to 9.7%. Ex vivo analysis showed an alteration of the resident cones in the lesioned area that was quantified within the PCAs and showed a significant loss by 1 day after LIP (57% survival of cones) that progressed until 14 days, when cone survival represented approximately 15% of the original values. In parallel, the microglial study revealed a clear activation of Iba-1^+^ cells in the OS layer which accumulated in the lesioned area and acquired an ameboid morphology, already apparent by 3 days after LIP which gradually relaxed to a more branching pattern at 7–14 days after LIP. It is remarkable that between 14 and 30 days after LIP, autofluorescent materials appeared within the lesion area.

### 3.1. LIP Induces a Focal Alteration in the Superior-Temporal Retina Resulting in Thinning of the Outer Retinal Layers within the Lesion Area

The SD-OCT study showed an alteration in the superior-temporal region of the retina that, as it is typical of this model, was well defined and consistently located in all the studied retinas. This lesion progressed with time, producing a decrease in retinal thickness in the central part of the lesion, that was more marked in the outer retina. Although this alteration has been characterised in previous studies of both diffuse [22,33,34] and focal [15,18] blue LED phototoxic injury, our present work shows for the first time the effects over a long period of time, 30 days after LIP. Previous studies [24] have examined the effect of long-term ultraviolet LED focal damage on retinal thickness in pigmented C57Bl/6 mice, and although the exposure parameters were different, the authors report a similar retinal thinning with greater reduction of the outer retina from 2 to 12 weeks post-phototoxicity [24]. Thus, it is conceivable that the phototoxic focal lesion starts up a dynamic process that evolves with time, thereby making it an appropriate model for the study of neuroprotective therapies [15,18,33,34]. 

### 3.2. Activation of Iba-1^+^ Reactive Monocytic Cells in the Focal Area of Injury Affecting the Cone Population

Focal phototoxic injury causes progressive S-cone loss within the lesioned area that has been recently described by our group both in rat [15] and mouse [15,18,33,34]. Our present studies in adult albino rat are somewhat similar to our previous studied by Ortín-Martínez et al. (2014) [15]. Indeed, our studies show a loss of S cones by 7 days that is comparable [15], thus confirming that the type of focal lesion induced by LIP is homologous. There is, however, an important difference between the methodology employed in the study of Ortín-Martínez et al. (2014) and that of our present study, and this is the duration of LED exposure; 10 secs for the Ortín-Martínez et al.’s (2014) study and 20 secs for the present study. Such a difference may explain why we find a progressive loss of S cones beyond 7 days, which was not observed in our previous report [15].

A recent study of the effects of LIP in albino mice [18] showed that cone loss was accompanied by an activation of microglial cells delineating the focal lesioned area. These cells reach the area of injury to activate immune resistance and modulated their morphology over time post-injury, from a more amoeboid-activated form, with a peak at 3 days, to a more branched-relaxed one [18]. This short-term effect has also been observed in a model of blue LED phototoxicity in pigmented mice [29,33]. However, this is the first study to analyse the long-term status of microglia following blue LED-induced phototoxicity. The present study shows the presence of autofluorescent material in the OS layer (yellow in Figure 2G–H’) which is detected with both the green (488 nm) and red (564 nm) filters of the fluorescence microscope, and which starts to appear at 14 days after LIP and is more evident at 30 days. Previous studies have postulated that this autofluorescent material present in the OS layer may be derived from the oxidation of OS from photoreceptors which results in faster phagocytosis, and an accumulation of lipofuscin-like material [33]. This aggregation could interfere with their ability to remove cellular debris and may be one of the factors in the degenerative mechanism of overexposure to light.

### 3.3. Limitations of the Present Study

One of the major limitations of the present study is that the status of the L cones in these retinas could not be assessed because they were not compatible with the other two antibodies used, according to the protocols established by our laboratory. This protocol establishes a routine detection of L-cone outer segments with the use of the primary antibody anti-opsin red/green (Chemicon-Millipore Iberica, Madrid, Spain) made in rabbit, as well as the antibody used to detect Iba-1^+^ cells (Abcam, Cambridge, UK). The long-term study of L cones and rhodopsin, to assess the behaviour of rods in response to this insult, could give us more information on the dynamic degenerative process that photoreceptors follow.

Although it is important to decipher the origin of activated microglia (Iba-1 labelled cells within the lesioned area), our current studies cannot reliably define whether activated microglia correspond to local microglia, to microglia that migrates from neighbouring regions of the retina, or even from invading blood monocyte cells, and thus future studies are needed to resolve this question.

### 3.4. Concluding Remarks

This study shows for the first time the effects of focal blue LED phototoxic injury on retinal thickness. The in vivo measurements show that this lesion appears visible 24 h after induction of the lesion and progressively reduces the thickness of the outer retinal layers until 30 days after induction, when the thickness of the outer retinal layers is reduced by approximately 90%, and the total thickness is reduced by approximately 50%. The ex vivo study shows a progressive reduction in the S-cone population that is accompanied by an activation of microglia that changes its morphology to an activated state with a peak at 3 days and gradually relaxes to a more branched form and leads to visible autofluorescent deposits at 14–30 days after LIP.

## 4. Materials and Methods

### 4.1. Animal Handling

To carry out these experiments we used 42 adult female Sprague Dawley (SD) albino rats (180–200 g). Rats were obtained from the Charles Rivers Laboratories (L’Arbresle, France) and housed in the animal facilities of the University of Murcia (UM) in temperature and light controlled rooms (12 h light/dark cycle) with food and water “ad libitum”. Animal manipulations followed the ARVO and European Union guidelines for the use of animals in research and were approved by the Ethical and Animal Studies Committee (University of Murcia protocol numbers A13171103, A13170110, and A13170111) and no manipulation that could involve pain or suffering for the animal was performed without adequate analgesia.

For photoexposure SD-OCT scanning, rats were anaesthetized with a mixture of xylazine (20 mg/mL, Xilagesic^®^, Laboratorios Calier, Barcelona, Spain) and ketamine (50 mg/mL, Imagene^®^ 50 mg/mL; Merial Laboratorios, S.A.U. Sant Cugat del Vallès, Barcelona, Spain) administered intraperitoneally (ip). An ocular ointment (Tobrex^®^, Alcon-Cusí, S.A., El Masnou, Barcelona, Spain) was instilled to prevent corneal desiccation in anaesthetized animals.

### 4.2. Light-Emitting Diode (LED)-Induced-Phototoxicity (LIP)

To perform the blue LIP model in albino rats, we followed the protocols previously established by our laboratory [15,18]. Briefly, in dark-adapted rats for at least 10–12 h, pupillary mydriasis of the left experimental eyes was induced by instillation of tropicamide (Tropicamida 1%^®^; Alcon-Cusí, S.A., El Masnou, Barcelona, Spain) and photoexposure was performed using a blue LED (emission spectrum 390–410 nm; catalogue number 454–4405; Kingbright Elec. Co., Taipei, Taiwan) located at 1 mm from the corneal apex and connected to a computer to control the duration of exposure (20 s) and light intensity (200 lux). To ensure the repeatability of the model, light intensity was measured with a luxometer (light meter TES-1330; TES Electrical Electronic Corp., Taipei, Taiwan) and the heads of the animals were maintained immobilised with a head holder during the whole process. Because light passes through the ocular optical media (cornea and lens) before reaching the retina, the estimated transmittance in rats was ≈78% for blue light (400 nm) [31]. Therefore, we assume that, under our experimental conditions, approximately 80% of the energy provided by the LED reaches the retina.

### 4.3. Spectral Domain Optical Coherence Tomography (SD-OCT)

As previously described [15,18], the in vivo study of retinal thickness monitoring after photoexposure in anaesthetised rats with dilated pupils was performed using a SD-OCT device (Spectralis; Heidelberg Engineering, Heidelberg, Germany). The LED photoexposure caused a focal alteration in the superior-temporal retina and its location and evolution was analysed previously and at 1, 3, 5, 7, 14, and 30 days by acquisition of fundus images and optical sections from the centre of the lesion. To measure the total retinal thickness (from the fibre layer to the retinal pigment epithelium) and the outer retinal thickness (from the outer plexiform layer to the retinal pigment epithelium) at the centre of the lesion, we used the average of three calliper measurements provided directly by the device software.

### 4.4. Tissue Processing

For sacrifice, rats were deeply anaesthetised by an overdose of sodium pentobarbital (1:1 in saline; Dolethal Vetoquinol, S.A., Lure, France) and perfused transcardially with saline and 4% paraformaldehyde in 0.1 M phosphate buffer at 1, 3, 5, 7, 14, or 30 days (*n* = 6 per group) after LIP. Once perfused, the eyes of each animal were removed from their orbits and the retinas were dissected as flattened wholemounts using a surgical microscope following protocols previously established by our laboratory [15,18,35].

### 4.5. Inmunohistofluorescence

Immunodetection of S-opsin^+^OS using S-opsin antibody (goat anti-OPN1SW; 1:1000; Santa Cruz Biotechnologies, Heidelberg, Germany) and reactive-Iba-1^+^ monocytic cells using Iba-1 antibody (1:500; rabbit anti-Iba-1, Abcam, Cambridge, UK) detected with Alexa Fluor-594 donkey anti-goat and Alexa Fluor-488 donkey anti-rabbit (1:500; IgG (H + L), Molecular Probes Invitrogen, Barcelona, Spain), respectively, was performed on whole mounts following previously described protocols [15,18,25]. 

### 4.6. Retinal Analysis

All retinal wholemounts were examined and photographed with a fluorescence microscope (Axioscop 2 Plus; Zeiss, Jena, Germany) equipped with a digital camera (ProgRes C10; Jenoptic, Jena, Germany) and a computer driven motorized stage (ProScan H128 Series; Prior Scientific Instruments, Cambridge, UK) controlled by Image-Pro Plus software (IPP 5.1 for windows; Media Cybernetics, Silver Spring, MD, USA), following protocols that are standard in our laboratory [15,25,35]. Photomontages of wholemounts were constructed from 90 consecutive frames captured side by side and, if necessary, images were further processed with a graphics editing programme (Adobe Photoshop CS 8.0.1; Adobe Systems, Inc., San Jose, CA, USA). 

### 4.7. Definition of a Predetermined Fixed-Size Circular Area (PCA)

Our previous studies using a similar blue LED induced retinal phototoxicity have documented that the LIP results in a small focal lesion almost devoid of S and L cones, located within the superior-temporal retina, that involves a minute portion of the entire retinal area (e.g., ≈1.3% for the rat [15] and ≈1.3% for the mouse [33]). Such a small lesion explains why total counts of S or L cones did not differ between the photoexposed and their fellow contralateral non-photoexposed retinas. Thus, to quantify cone loss within the focal area of lesion, or to examine the response of microglial cells, it was necessary to restrict cone counts and retinal inspection to a small PCA are (with a radius of 1.3 mm) centred in the middle of the lesion, of all experimental retinas as well as in the equivalent region of the fellow non-photoexposed contralateral retina, as described [15,18,32].

Thus, to study the long-term effects of LIP on the population of S-opsin^+^OS, these were automatically quantified within the PCA following previously described protocols [15,18,34]. S-cone counts were compared with our previous studies to ensure an analogous lesion [15]. For each experimental animal, these counts were obtained from the left retina and a corresponding region in its fellow non-photoexposed contralateral untouched right retina. Higher power images of Iba-1^+^cells and S cones^+^OS located within the lesioned were obtained and photographed in whole mounts using a Leica SP8 confocal microscope (20×, 40×, or 63×, Leica Microsytems, Wetzlar, Germany).

### 4.8. Statistical Analysis

Statistical analysis was done using GraphPad Prism^®^ for windows (Version 5.01; GraphPad Software, San Diego, CA, USA). One-way ANOVA test was used to compare retinal thicknesses and S-opsin^+^OS populations at different time points after LIP. Data is shown as mean ± standard deviations (mean ± SD) and differences were considered significant when *p* < 0.05.

## Figures and Tables

**Table 1:** Total number of S-opsin^+^OS within PCAs.

Rat	RE (1 Day)	RE (30 Days)	1 Day	3 Days	7 Days	14 Days	30 Days
1	2018	2890	1350	913	1008	442	466
2	2680	1965	1901	1334	788	260	561
3	2709	2589	770	765	359	571	453
4	1803	2194	1479	927	1171	219	393
5	2874	2239	1424	942	644	314	371
6	2749	2075	1287	1025	797	356	427
Mean	2321	2477	1369 *	937	795	361 ^§^	445
SD	435	357	364	213	283	129	67
RE Pooled mean	2398						
RE Pooled SD	388						

Total number of S-opsin^+^OS within the PCAs of the non-photoexposed right PCAs (RE) of the groups analysed at 1 and 30 days and of the experimental retinas photoexposed analysed at 1, 3, 7, 14, or 30 days. SD: Standard Deviation. * one-way ANOVA: *p* = 0.001 when compared with RE. ^§^ one-way ANOVA: *p* = 0.002 when compared with 3 days.

## Data Availability

The data presented in this study are available on request from the corresponding author.

## References

[1] Klein R., Cruickshanks K.J., Nash S.D., Krantz E.M., Nieto F.J., Huang G.H., Pankow J.S., Klein B.E. (2010). The prevalence of age-related macular degeneration and associated risk factors. Arch. Ophthalmol..

[2] Friedman D.S., O’Colmain B.J., Munoz B., Tomany S.C., McCarty C., De Jong P.T., Nemesure B., Mitchell P., Kempen J. (2004). Eye Diseases Prevalence Research, G., Prevalence of age-related macular degeneration in the United States. Arch. Ophthalmol..

[3] Garcia-Layana A., Cabrera-Lopez F., Garcia-Arumi J., Arias-Barquet L., Ruiz-Moreno J.M. (2017). Early and intermediate age-related macular degeneration: Update and clinical review. Clin. Interv. Aging.

[4] Lambert N.G., ElShelmani H., Singh M.K., Mansergh F.C., Wride M.A., Padilla M., Keegan D., Hogg R.E., Ambati B.K. (2016). Risk factors and biomarkers of age-related macular degeneration. Prog. Retin. Eye Res..

[5] Alaimo A., Linares G.G., Bujjamer J.M., Gorojod R.M., Alcon S.P., Martinez J.H., Baldessari A., Grecco H.E., Kotler M.L. (2019). Toxicity of blue led light and A2E is associated to mitochondrial dynamics impairment in ARPE-19 cells: Implications for age-related macular degeneration. Arch. Toxicol..

[6] Klein R., Klein B.E., Knudtson M.D., Meuer S.M., Swift M., Gangnon R.E. (2007). Fifteen-year cumulative incidence of age-related macular degeneration: The Beaver Dam Eye Study. Ophthalmology.

[7] Sui G.Y., Liu G.C., Liu G.Y., Gao Y.Y., Deng Y., Wang W.Y., Tong S.H., Wang L. (2013). Is sunlight exposure a risk factor for age-related macular degeneration? A systematic review and meta-analysis. Br. J. Ophthalmol..

[8] Di Pierdomenico J., Garcia-Ayuso D., Pinilla I., Cuenca N., Vidal-Sanz M., Agudo-Barriuso M., Villegas-Perez M.P. (2017). Early Events in Retinal Degeneration Caused by Rhodopsin Mutation or Pigment Epithelium Malfunction: Differences and Similarities. Front. Neuroanat..

[9] Di Pierdomenico J., Scholz R., Valiente-Soriano F.J., Sanchez-Migallon M.C., Vidal-Sanz M., Langmann T., Agudo-Barriuso M., Garcia-Ayuso D., Villegas-Perez M.P. (2018). Neuroprotective Effects of FGF2 and Minocycline in Two Animal Models of Inherited Retinal Degeneration. Investig. Ophthalmol. Vis. Sci..

[10] Garcia-Ayuso D., Salinas-Navarro M., Agudo M., Cuenca N., Pinilla I., Vidal-Sanz M., Villegas-Perez M.P. (2010). Retinal ganglion cell numbers and delayed retinal ganglion cell death in the P23H rat retina. Exp. Eye Res..

[11] LaVail M.M., Nishikawa S., Steinberg R.H., Naash M.I., Duncan J.L., Trautmann N., Matthes M.T., Yasumura D., Lau-Villacorta C., Chen J. (2018). Phenotypic characterization of P23H and S334ter rhodopsin transgenic rat models of inherited retinal degeneration. Exp. Eye Res..

[12] Pinilla I., Fernandez-Sanchez L., Segura F.J., Sanchez-Cano A.I., Tamarit J.M., Fuentes-Broto L., Eells J.T., Lax P., Cuenca N. (2016). Long time remodeling during retinal degeneration evaluated by optical coherence tomography, immunocytochemistry and fundus autofluorescence. Exp. Eye Res..

[13] LaVail M.M. (1981). Photoreceptor characteristics in congenic strains of RCS rats. Investig. Ophthalmol. Vis. Sci..

[14] Garcia-Ayuso D., Salinas-Navarro M., Agudo-Barriuso M., Alarcon-Martinez L., Vidal-Sanz M., Villegas-Perez M.P. (2011). Retinal ganglion cell axonal compression by retinal vessels in light-induced retinal degeneration. Mol. Vis..

[15] Ortin-Martinez A., Valiente-Soriano F.J., Garcia-Ayuso D., Alarcon-Martinez L., Jimenez-Lopez M., Bernal-Garro J.M., Nieto-Lopez L., Nadal-Nicolas F.M., Villegas-Perez M.P., Wheeler L.A. (2014). A novel in vivo model of focal light emitting diode-induced cone-photoreceptor phototoxicity: Neuroprotection afforded by brimonidine, BDNF, PEDF or bFGF. PLoS ONE.

[16] Riccitelli S., Di Paolo M., Ashley J., Bisti S., Di Marco S. (2021). The Timecourses of Functional, Morphological, and Molecular Changes Triggered by Light Exposure in Sprague-Dawley Rat Retinas. Cells.

[17] Zhang C., Lei B., Lam T.T., Yang F., Sinha D., Tso M.O. (2004). Neuroprotection of photoreceptors by minocycline in light-induced retinal degeneration. Investig. Ophthalmol. Vis. Sci..

[18] Valiente-Soriano F.J., Ortin-Martinez A., Di Pierdomenico J., Garcia-Ayuso D., Gallego-Ortega A., Miralles de Imperial-Ollero J.A., Jimenez-Lopez M., Villegas-Perez M.P., Wheeler L.A., Vidal-Sanz M. (2019). Topical Brimonidine or Intravitreal BDNF, CNTF, or bFGF Protect Cones Against Phototoxicity. Transl. Vis. Sci. Technol..

[19] Jaadane I., Villalpando Rodriguez G.E., Boulenguez P., Chahory S., Carre S., Savoldelli M., Jonet L., Behar-Cohen F., Martinsons C., Torriglia A. (2017). Effects of white light-emitting diode (LED) exposure on retinal pigment epithelium in vivo. J. Cell Mol. Med..

[20] Kim G.H., Kim H.I., Paik S.S., Jung S.W., Kang S., Kim I.B. (2016). Functional and morphological evaluation of blue light-emitting diode-induced retinal degeneration in mice. Graefes Arch. Clin. Exp. Ophthalmol..

[21] Kuse Y., Ogawa K., Tsuruma K., Shimazawa M., Hara H. (2014). Damage of photoreceptor-derived cells in culture induced by light emitting diode-derived blue light. Sci. Rep..

[22] Nakamura M., Kuse Y., Tsuruma K., Shimazawa M., Hara H. (2017). The Involvement of the Oxidative Stress in Murine Blue LED Light-Induced Retinal Damage Model. Biol. Pharm. Bull..

[23] Nakamura M., Yako T., Kuse Y., Inoue Y., Nishinaka A., Nakamura S., Shimazawa M., Hara H. (2018). Exposure to excessive blue LED light damages retinal pigment epithelium and photoreceptors of pigmented mice. Exp. Eye Res..

[24] Meer A.V., Berger T., Muller F., Foldenauer A.C., Johnen S., Walter P. (2020). Establishment and Characterization of a Unilateral UV-Induced Photoreceptor Degeneration Model in the C57Bl/6J Mouse. Transl. Vis. Sci. Technol..

[25] Ortin-Martinez A., Jimenez-Lopez M., Nadal-Nicolas F.M., Salinas-Navarro M., Alarcon-Martinez L., Sauve Y., Villegas-Perez M.P., Vidal-Sanz M., Agudo-Barriuso M. (2010). Automated quantification and topographical distribution of the whole population of S- and L-cones in adult albino and pigmented rats. Investig. Ophthalmol. Vis. Sci..

[26] Ortin-Martinez A., Nadal-Nicolas F.M., Jimenez-Lopez M., Alburquerque-Bejar J.J., Nieto-Lopez L., Garcia-Ayuso D., Villegas-Perez M.P., Vidal-Sanz M., Agudo-Barriuso M. (2014). Number and distribution of mouse retinal cone photoreceptors: Differences between an albino (Swiss) and a pigmented (C57/BL6) strain. PLoS ONE.

[27] Geiger P., Barben M., Grimm C., Samardzija M. (2015). Blue light-induced retinal lesions, intraretinal vascular leakage and edema formation in the all-cone mouse retina. Cell Death Dis..

[28] Song J.A., Choi C.Y. (2019). Effects of blue light spectra on retinal stress and damage in goldfish (Carassius auratus). Fish. Physiol. Biochem..

[29] Jaadane I., Boulenguez P., Chahory S., Carre S., Savoldelli M., Jonet L., Behar-Cohen F., Martinsons C., Torriglia A. (2015). Retinal damage induced by commercial light emitting diodes (LEDs). Free Radic. Biol. Med..

[30] Xu S., Zhang P., Zhang M., Wang X., Li G., Xu G., Ni Y. (2021). Synaptic changes and the response of microglia in a light-induced photoreceptor degeneration model. Mol. Vis..

[31] Chang S.W., Kim H.I., Kim G.H., Park S.J., Kim I.B. (2016). Increased Expression of Osteopontin in Retinal Degeneration Induced by Blue Light-Emitting Diode Exposure in Mice. Front. Mol. Neurosci..

[32] Krigel A., Berdugo M., Picard E., Levy-Boukris R., Jaadane I., Jonet L., Dernigoghossian M., Andrieu-Soler C., Torriglia A., Behar-Cohen F. (2016). Light-induced retinal damage using different light sources, protocols and rat strains reveals LED phototoxicity. Neuroscience.

[33] Miralles de Imperial-Ollero J.A., Gallego-Ortega A., Norte-Muñoz M., Di Pierdomenico J., Valiente-Soriano F.J., Vidal-Sanz M. (2021). An in vivo model of focal light emitting diode-induced cone photoreceptor phototoxicity in adult pigmented mice: Protection with bFGF. Exp. Eye Res..

[34] Valiente-Soriano F.J., Di Pierdomenico J., Garcia-Ayuso D., Ortin-Martinez A., Miralles de Imperial-Ollero J.A., Gallego-Ortega A., Jimenez-Lopez M., Villegas-Perez M.P., Becerra S.P., Vidal-Sanz M. (2020). Pigment Epithelium-Derived Factor (PEDF) Fragments Prevent Mouse Cone Photoreceptor Cell Loss Induced by Focal Phototoxicity In Vivo. Int. J. Mol. Sci..

[35] Valiente-Soriano F.J., Garcia-Ayuso D., Ortin-Martinez A., Jimenez-Lopez M., Galindo-Romero C., Villegas-Perez M.P., Agudo-Barriuso M., Vugler A.A., Vidal-Sanz M. (2014). Distribution of melanopsin positive neurons in pigmented and albino mice: Evidence for melanopsin interneurons in the mouse retina. Front. Neuroanat..

